# Congress Statement on the Appraisal and Prospects for Person-Centered Medicine in Croatia

**DOI:** 10.3325/cmj.2014.55.83

**Published:** 2014-02

**Authors:** Veljko Đorđević, Marijana Braš, Lovorka Brajković

**Affiliations:** Centre for Palliative Medicine, Medical Ethics and Communication Skills, School of Medicine University of Zagreb, Zagreb, Croatia

To address the Appraisal and Prospects for Person-Centered Medicine in Croatia, members of the Local Organizing Group met during the First International Congress of the ICPCM in Zagreb, Croatia on November 8th, 2013.

Starting at the Zagreb School of Public Health in 1927, the promotion of person-centered medicine and people-centered health care, based on the teachings and legacy of professor Andrija Štampar, has been occurring in Croatia for decades. Professor Andrija Štampar, one of the founders of the WHO and a representative at its first general meeting, considered by many as the father of public health, pioneered various public health projects in Croatia and abroad. He was, above all, a great educator who inspired many of his students and the general public alike, to promote the concept of health for all and the importance of health-related activities within the community, with emphasis on the responsibility of each individual for his/her own health. Croatia is a country with a long history of patient associations, as well as one with an array of public health projects recognized worldwide. Recently, a group of enthusiasts gathered here in Croatia to undertake the creation of a variety of projects related to the development of person-centered medicine, with the help of leading academic associations along with the President of the Republic, Professor Ivo Josipovic. Among other things, a multitude of programs were introduced to the curriculum of the University of Zagreb School of Medicine at the undergraduate and post-graduate levels, with the aim of perfecting the curriculum to reflect the concepts of person-centered medicine. Various symposiums, workshops, media activities, collaboration with patient associations within Croatia, post-graduate seminars, and national conferences about psychiatry and person-centered medicine were held in Zagreb in 2012, and an English-language book was published with the collaboration of over a hundred authors from Croatia and abroad entitled “The person in medicine and healthcare - from bench to bedside to community”. Croatian representatives were actively involved in a variety of activities of the International College of Person-Centered Medicine (ICPCM) and organized the First International Congress of the International College of Person-Centered Medicine in November 2013.

With that in mind, we believe that Croatia could contribute significantly to the development of person-centered medicine and people-centered health care, within Croatia and abroad. We intend to continue the activities promoted by ICPCM and its associated institutions. Therefore, we resolve to undertake a project entitled “The International Academy for Person-Centered Medicine and People-Centered Healthcare”. The goal of this project is initiate various multimedia educational activities for health care professionals, patient associations, and the general public with the intention of promoting fundamental provisions and principles of person-centered medicine, people-centered health care, and promote the culture of health and wellness. Our plan is to submit a declaration of intent to the ICPCM for collaboration on this project. We are convinced that, together, we can make our community better and healthier. There is no future without a clear understanding of the past, the future is being built today, and today is yesterday’s tomorrow, so we have to continue working together and learning from one another.

